# The Analytical Validity of Stride Detection and Gait Parameters Reconstruction Using the Ankle-Mounted Inertial Measurement Unit Syde^®^

**DOI:** 10.3390/s24082413

**Published:** 2024-04-10

**Authors:** Mona Michaud, Alexandre Guérin, Marguerite Dejean de La Bâtie, Léopold Bancel, Laurent Oudre, Alexis Tricot

**Affiliations:** 1 Sysnav, 27200 Vernon, France; mona.michaud@ens-paris-saclay.fr (M.M.); alexandre.guerin@universite-paris-saclay.fr (A.G.); leopold.bancel@sysnav.fr (L.B.);; 2 Université Paris Saclay, Université Paris Cité, ENS Paris Saclay, CNRS, SSA, INSERM, Centre Borelli, 91190 Gif-sur-Yvette, France; 3 DataShape, Inria Saclay Ile-de-France, 91120 Palaiseau, France; 4 Laboratoire de Mathématiques d’Orsay, Université Paris-Saclay, CNRS, 91405 Orsay, France

**Keywords:** IMU, wearable, analytical validation, motion capture, gait monitoring, controlled environment

## Abstract

The increasing use of inertial measurement units (IMU) in biomedical sciences brings new possibilities for clinical research. The aim of this paper is to demonstrate the accuracy of the IMU-based wearable Syde^®^ device, which allows day-long and remote continuous gait recording in comparison to a reference motion capture system. Twelve healthy subjects (age: 23.17 ± 2.04, height: 174.17 ± 6.46 cm) participated in a controlled environment data collection and performed a series of gait tasks with both systems attached to each ankle. A total of 2820 strides were analyzed. The results show a median absolute stride length error of 1.86 cm between the IMU-based wearable device reconstruction and the motion capture ground truth, with the 75th percentile at 3.24 cm. The median absolute stride horizontal velocity error was 1.56 cm/s, with the 75th percentile at 2.63 cm/s. With a measurement error to the reference system of less than 3 cm, we conclude that there is a valid physical recovery of stride length and horizontal velocity from data collected with the IMU-based wearable Syde^®^ device.

## 1. Introduction

Motion analysis in healthcare is a standard tool that is essential in medicine and clinical research [1]. Reduced or impaired mobility occurs in many chronic health conditions, in the natural aging process, and is often associated with reduced quality of life, increased risk of falls, and mortality [2,3,4]. It is described as a meaningful criterion in neuromuscular [5,6] or neurological [2,3,7] diseases. Indeed, gait speed, referred to as the “6th vital sign of health” [8], is an informative and commonly used measure in the clinical assessment of patients [9]. Lower gait speeds are observed in the most clinically affected patients [2,3] and in older patients [4].

Quantifying patients’ mobility at the lower limb level is a key challenge that needs to be addressed in order to obtain an objective assessment of their clinical evolution. Current practice in measuring mobility includes patient-reported outcomes, objective clinical assessments (using standard walking scores), and subjective clinical assessments (using standard criteria) [10]. A quantitative approach using technologies such as motion capture systems, which permit the precise measurement of positions by triangulation, is possible. However, this technique is large, expensive, and difficult to use. An interesting alternative is inertial measurement units (IMUs), which have been used for the past twenty years. It has been an inexpensive, non-invasive, and user-friendly technology for studying motion occurring during physical activities [11]. This technology is widely used in controlled environments, hospitals, and academic laboratories [12], but can also be used in uncontrolled environments to measure real-world gait parameters. Experiments in controlled settings have the main advantage of ensuring accurate data acquisition and annotation, thus providing directly usable gait metrics through spatiotemporal feature computation [7]. However, when performed in a controlled environment, gait assessment provides snapshot measurements that may not accurately reflect the exact patient’s condition and may have the following limitations:

- High variability of data depending on the state and motivation of the participant at the time of data collection;
- Access to a fixed number of activities chosen by the experimenter;
- A white coat [13] and Hawthorne effect [14] due to experimental conditions.

For example, Shema-Shiratzky et al. (2020) [15] observed a lower gait speed in patients observed in an uncontrolled environment rather than in those who participated in the data collection in the laboratory and, conversely, the opposite tendency was observed in the control population. It then justifies the need for real-world digital gait clinical outcomes in research and clinical trials.

The increasing use of biomedical technologies in healthcare over the last decade has enabled real-world remote monitoring and generation of digital mobility outcomes such as spatiotemporal (speed), temporal (pace, step time, swing time), and spatial (stride length, width) gait characteristics that quantify real-world walking and may be more reliable than gait assessment in a controlled environment [16,17,18]. Therefore, there is a growing interest in developing real-world digital mobility outcomes to quantify patients’ natural gait and ensure the quality, reliability, and validity of the development to limit the fragmentation and difficulty of the assessment of the method.

**Figure 1:**
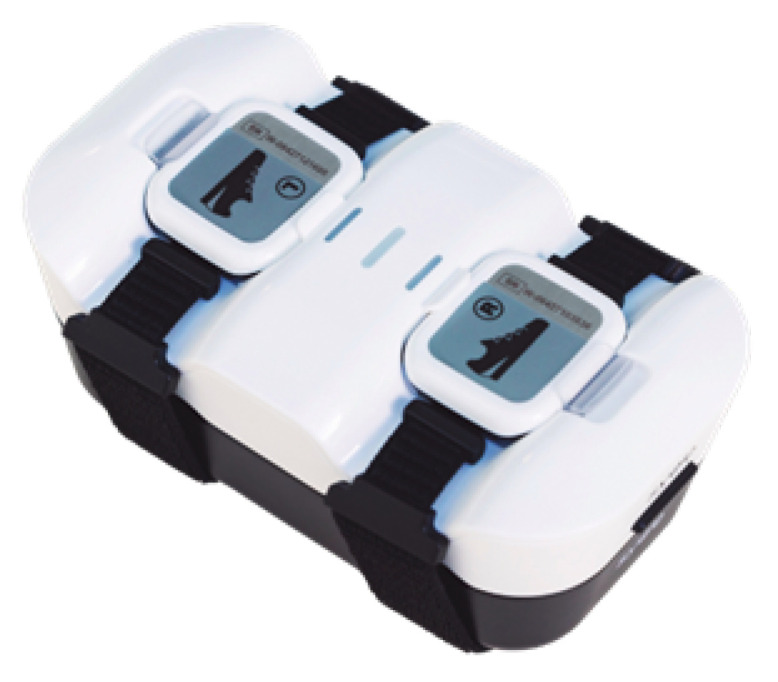
IMU-based wearable Syde^®^ device station with its two sensors docked.

**Figure 2:**
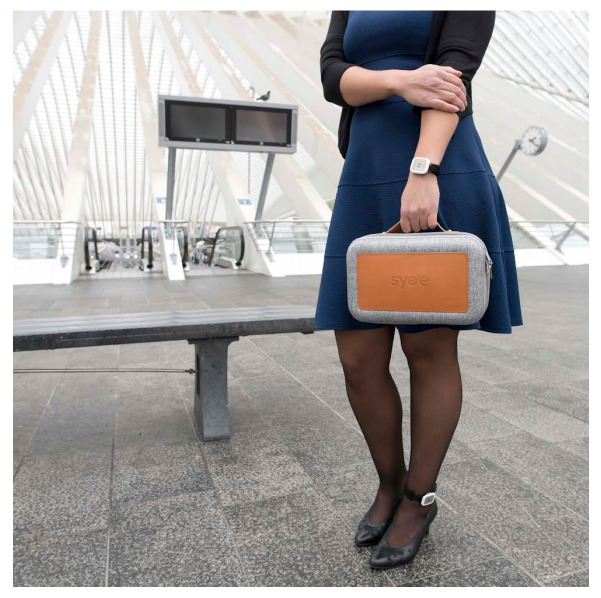
IMU-based wearable Syde^®^ sensors being worn on the left wrist and ankle.

The Syde^®^, an IMU-based wearable device that can be used in an uncontrolled environment, is one of these evolving biotechnologies (Figure 1) that, like its first-generation predecessor, the ActiMyo^®^ [6,19], measures the patient’s movement in a real-life environment. This device consists of two IMUs, including a high-precision triaxial accelerometer, gyrometer, magnetometer, and barometer, measuring linear acceleration, angular velocity, magnetic field of motion (in all directions), and barometric altitude, all within the body frame of the device. Dedicated algorithms capture the limb trajectory, enabling high-fidelity 3D step reconstruction from which multiple-step parameters are derived. These wearable devices are being used in numerous clinical trials in neuromuscular pathologies, such as facioscapulohumeral muscular dystrophy [20], spinal muscular atrophy [21], and, most notably, in Duchenne Muscular Dystrophy [22] (Figure 2).

In addition, the wearable’s calculated features have been approved by health authorities. In 2019, the 95th centile of stride velocity (SV95C) was accepted by the European Medicines Agency for use as a secondary endpoint in pivotal trials in Duchenne Muscular Dystrophy (DMD) [23] and is currently being used in various clinical trials [24,25]. In July 2023, the SV95C digital outcome was qualified as a primary endpoint in clinical trials in ambulatory patients with Duchenne Muscular Dystrophy [26], allowing it to be used as a first-order measure of treatment efficacy. It became the first wearable-derived digital clinical outcome measure to be qualified as a primary endpoint by the European Medicines Agency. This work follows the “V3” digital clinical measure validation scheme described by the Digital Medicine Society, which has been adopted by the European Medical Agency. It requires sensor verification (sensor performance), the analytical validation of processing algorithms (ability to measure physiological metrics), and clinical validation (ability to identify, measure, or predict clinically meaningful functional states in a specified population and context of use). This study is, therefore, part of a long-term project to develop a digital mobility endpoint that can be used as a primary endpoint in clinical trials, such as SV95C in Duchenne Muscular Dystrophy [26].

The primary objective of this study is to perform an analytical validation in a controlled environment of the wearable device dedicated algorithms on stride length and horizontal velocity compared with the same features obtained with a motion capture reference. A gait data collection session was conducted with 12 healthy adult volunteers. The participants were fitted with an IMU-based wearable device on both ankles in a motion capture room, which was also equipped with markers to record motion. The participants were asked to perform predefined gait exercises in a controlled environment. The stride length and stride horizontal velocity for all exercises calculated using Sysnav’s proprietary algorithm were compared with those obtained using the motion capture setup.

This paper is structured in the following way. Section 2 provides details of the motion capture materials, the experimental protocol, and the analysis method. Section 3 presents the results of the stride detection, the feature distribution between the IMU-based wearable and the motion capture system, and the error distribution between the two systems. The results are discussed in Section 4. Finally, Section 5 concludes this article.

## 2. Materials and Methods

This section presents the gait outcomes of interest and the experimental setup used to measure them.

### 2.1. Motion Recording Materials

#### 2.1.1. IMU-Based Wearable Device

In this study, an IMU-based wearable device is attached to both ankles of each participant to record inertial data, which, after analysis, allows the reconstruction of the gait parameters of interest, namely the stride length and the horizontal stride velocity. The model used is Sysnav Syde^®^, which includes a high-precision triaxial accelerometer, triaxial gyroscope, barometer, and a temperature sensor, recording data at a sampling rate of around 100 Hz. This IMU-based wearable device continuously monitors the patient’s daily activities at home and outdoors for up to 14 h.

The data recorded by the sensors follow the processing pipeline below:

1. Raw data are written on the docking station after the sensors are reattached;
2. Station uploads the raw data to a remote secure cloud;
3. Usable data are extracted against the sensors’ calibration files;
4. Trajectory is estimated at the stride level from the calibrated inertial data;
5. Gait parameters are estimated from the reconstructed trajectory.

The gait parameters of interest in this study are the stride length and the mean horizontal velocity over a stride.

#### 2.1.2. Motion Capture

In this study, the motion capture is used as the ground truth to compare the stride reconstruction performed on the inertial data collected by the IMU-based wearable device.

Twelve infrared OptiTrack^®^ Prime 13 W cameras were mounted on tripods and arranged in an elliptical fashion to maximize the space available to perform the exercises defined in the protocol. Their placement must ensure optimal coverage while maximizing the number of cameras that can detect a given reflective marker present within the experimental area, which is formally the inside of their convex hull. The number of cameras and their placement are crucial settings because the motion capture process is based on the principle of triangulation: the 3D positions of the reflective markers can be computed if at least three cameras can see them. If a reflective marker is in the field of view of fewer than three cameras at any given time, the capture software is technically unable to determine the marker’s position, and the signal is temporarily lost.

The cameras were powered and wired using RJ45 cables to a network switch, which, in turn, was connected to a computer used to store and then analyze the collected data. The visual signals recorded by the cameras were processed by the OptiTrack^®^ Motive software (v1.10.0) to produce the usable position and attitude data at a sampling rate of 120 Hz.

The motion was recorded by the cameras using reflective markers. Markers are solid plastic spheres, 12.5 to 16 mm in diameter, covered with a gray reflective coating. The markers can be used separately or mounted together on a rigid body to reduce missing data due to obstructions.

The acquisition process is preceded by a calibration routine. Its purpose is to define an inertial reference frame for the measurements and to determine the relative and absolute positions of the camera within this frame. A calibration square with markers on each edge was placed on the ground to define the inertial frame. The position of the cameras was determined by moving a calibration wand around the field of view until satisfactory coverage was achieved. Finally, the software assessed the quality of the calibration and rated it on a scale from “very poor” to “exceptional”. In this study, calibrations were performed until a level of quality equivalent to “excellent” was achieved.

The technical specifications provided by OptiTrack indicate a three-dimensional accuracy of 0.3 mm, justifying its use as the ground truth in our experiment.

### 2.2. Experimental Protocol

The recording session was conducted in July 2022 and included 12 healthy adult volunteers (9 men, 3 women), age 23.17 ± 2.04 years, height 174.17 ± 6.46 cm, weight 65.08 ± 9.41 kg, and BMI 21.40 ± 2.29. Each participant received a written description of this study and signed an informed consent form. They explicitly agreed to the use of their data in this study. The exclusion criteria consisted of not including participants who had a current lower limb injury, so as not to interfere with thia study and its results, or who had undergone surgery within three months prior to this study.

The experimental session took place in an indoor motion capture room in Vernon, France.

An IMU-based wearable device was placed over the lateral malleolus of each ankle with a rigid body consisting of five to six reflective markers. A variable number of markers per rigid body was used to break any possible symmetry between the two rigid bodies, thus prophylactically preventing any rigid body inversion. In addition, the barycenter of each rigid body was positioned to coincide with the center of mass of the IMU-based portable device (Table 1).

**Figure 3:**
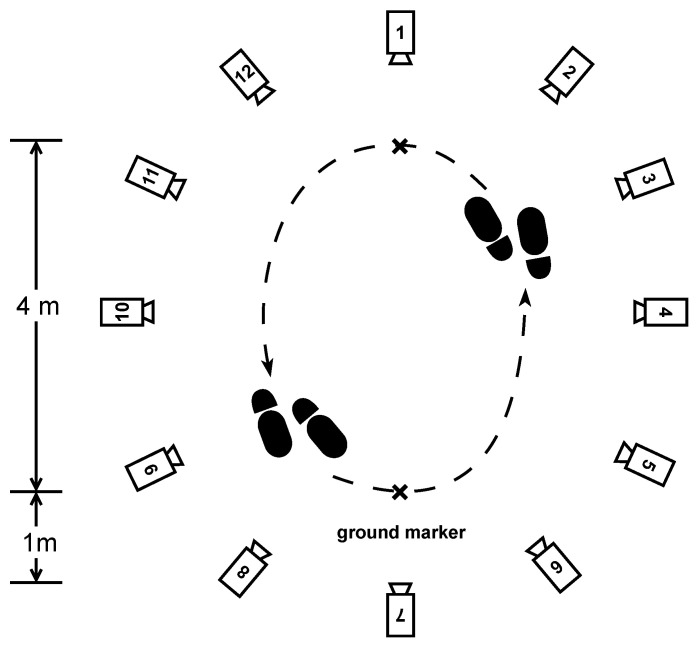
The experimental settings for the regular walking exercises (normal walk, fast walk, and dual-task walk). The twelve motion capture cameras are placed around the outside of the experimental area at a distance that allows the movements to be captured accurately.

**Figure 4:**
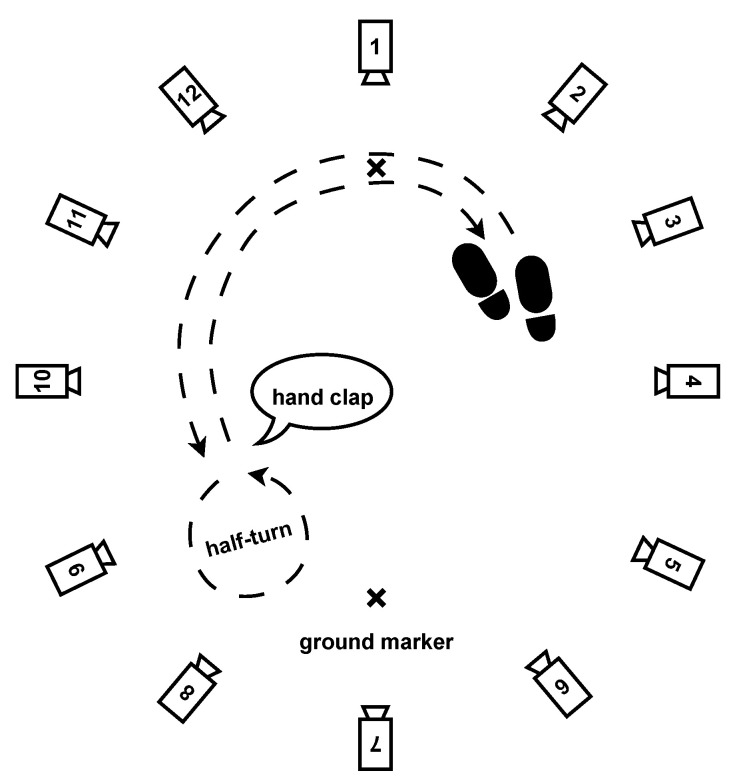
Experimental motion capture settings for half-turns exercise.

**Figure 5:**
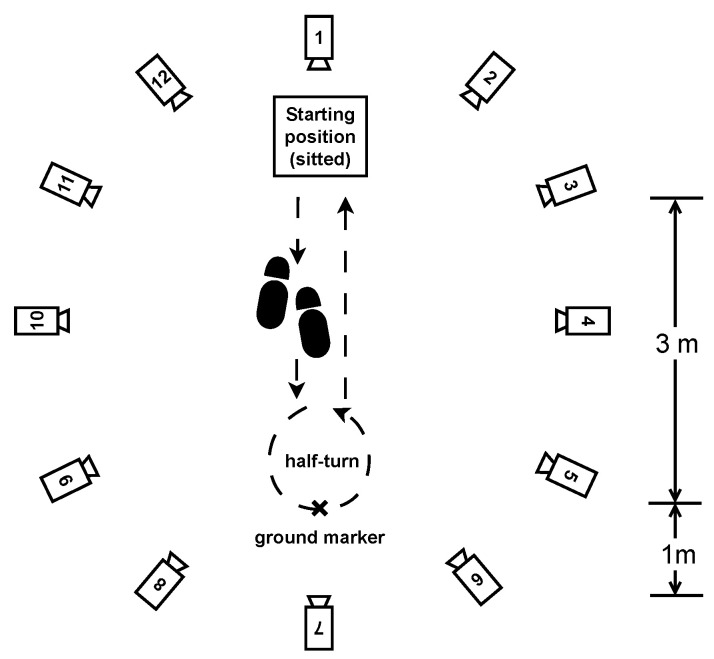
Experimental settings for timed up-and-go exercise.

The session included the following walking exercises:

- Normal walk: The participants were asked to walk a distance of 80 m. The walking route consisted of 10 two-way trips between two markers on the ground separated by 4 m, as shown in Figure 3. The instruction given to the participant was to “walk at your own comfortable walking pace”, as it is similarly seen in pace instructions in standard assessments of walking, such as the 10 MWT at a comfortable pace [27].
- Fast walk: The participants had the same setting and distance as in the normal walking exercise but were instructed to walk "as fast as they could" as is similarly seen in the instructions of gait pace in the 10 MWT [27].
- Dual-task walk: As inspired by a real-life condition, where "the multifaceted nature of the stimuli coming from the surrounding world requires responses that often imply the simultaneous performance of a motor and a cognitive task, i.e., dual-task" as presented by Pedullà et al. (2022) [28]. We choose to perform a dual task of walking and cognitive activity to change the patients’ walking behavior, such as walking speed, and get closer to real-life conditions, as shown in studies [29,30]: same setting, distance, and pace as in the normal walk exercise, but the participants were asked to perform a cognitive task at the same time. The task was to recite as many animal names as possible while walking.
- Half-turns: The same setting and pace as in the normal walk exercise, but this time, the participants had to walk for one minute and make a half-turn each time the experimenter clapped their hands. Seven hand claps occurred at predetermined and heterogeneously distributed times within one minute. See Figure 4.
- Timed up-and-go: Each participant started the exercise sitting in a chair, stood up, walked 3 m at their natural pace, turned, walked back to the chair, and sat down. This exercise was repeated three times with little or no rest. See Figure 5.

### 2.3. Analysis Method

#### 2.3.1. Gait Parameter Reconstruction from Inertial Data

**Figure 6:**
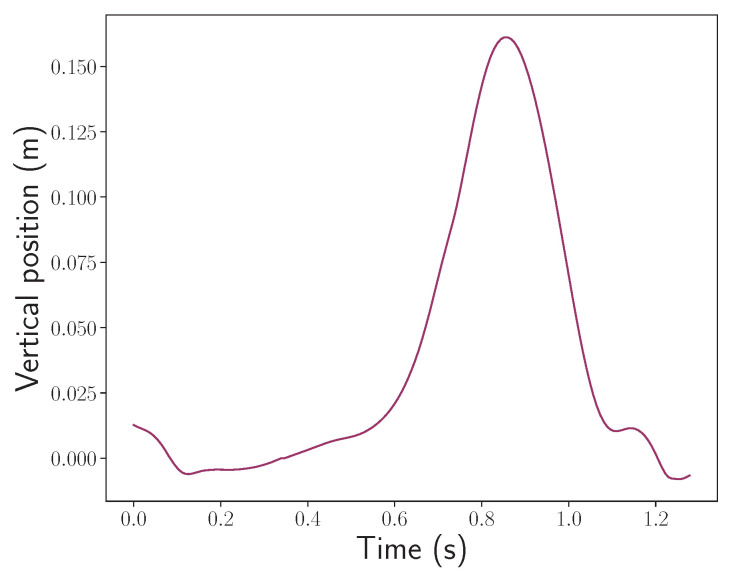
The vertical position motif of an ankle during a stride.

**Figure 7:**
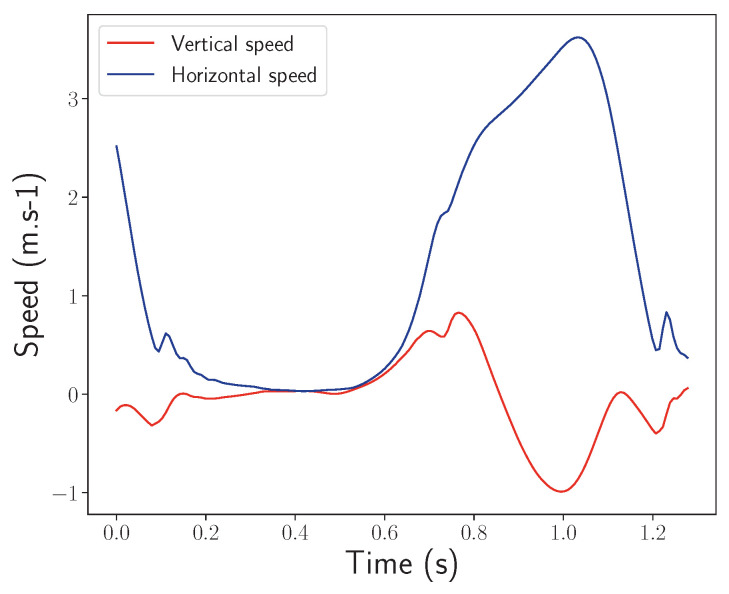
The horizontal and vertical speed motifs of an ankle during a stride.

The gait reconstruction algorithms used in this study fulfill two main objectives. The first one is to be able to integrate the sensor acquired data with the constraint of minimizing a quadratic time dependent drift and bias to obtain a physical trajectory in an inertial frame of reference. The second is to isolate steps based on the sole inertial data and then recover the length and average horizontal velocity during a step. The strides are isolated by identifying key gait phases in the reconstructed signals, which are passed through a segmentation algorithm based on a state machine. The position and velocity signals corresponding to a stride can be visualized in Figure 6 and Figure 7.

The underlying processes used are patented and registered [31,32].

#### 2.3.2. Motion Capture Data Processing

The physical setup of motion capture is described in the Section 2.1.2. The capture software performs full motion reconstruction from visual signals, directly outputting the positions and attitudes of markers and rigid bodies in a predefined inertial reference frame. The data are then synchronized in time with the IMU data by correlating the data from both devices. The synchronization accuracy is estimated to be the duration of two samples of IMU data, i.e., approximately 15 ms.

#### 2.3.3. Analytical Features Calculation

The two analytical features of interest in this study are stride length and average horizontal stride velocity. They are computed independently on both the limb inertial data and the motion capture data. The features are computed using inertial and optical motion reconstruction methods for all the strides performed by the twelve participants, from the data acquired on both ankles, for all the five exercises foreseen by the study protocol. To analyze the results, we consider the strides detected simultaneously on the inertial and motion capture signals for each exercise, limb, and participant.

## 3. Results

The results of the experiments defined in the study protocol and their statistical analysis are presented in this section.

### 3.1. Validation of Sensor-Based Stride Detection

A manual annotation of the 24 sensor-acquired recordings (12 participants, two wearing sides) was conducted to validate the performance of the inertial stride detection. Manual annotation consisted more precisely in segmenting strides with respect to the gyrometer and accelerometer signals, identifying strides by spotting key gait phases such as the heel strikes and the swing subphases. Afterward, the manually detected strides were compared to the inertial detection and tagged into three categories: the true positives, namely strides that have been correctly detected; the false negatives, which designate undetected strides; and the false positives, the wrongfully detected strides.

The entire data acquisition session produced a total of 6480 strides, out of which 6237 were actually retrieved with the use of the inertial stride detection algorithm. Among those 6237 strides, 6218 were correctly detected and 19 were false positives, representing on average less than one stride per file, or 0.3% of the total number of detected strides. As a corollary, the inertial segmentation missed 262 strides, representing 4.0% of the total existing number of strides.

**Figure 8:**
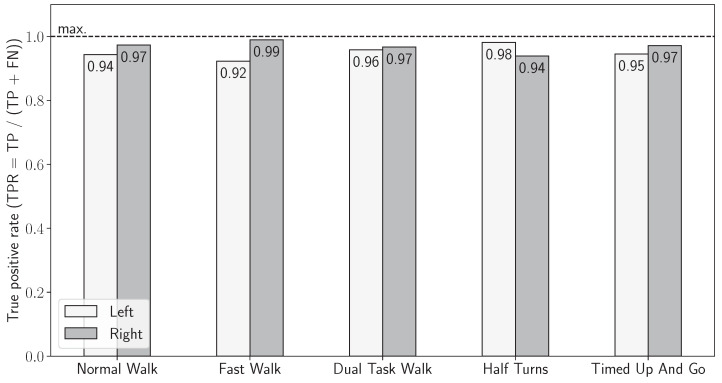
The true positive rate of the sensor-based stride detection by side and by exercise.

The sensor-based stride detection yielded excellent results when looking at each exercise individually, as shown in Figure 8 where the true positive rate of the method is displayed by exercise and side. This preliminary validation of the sensor-based segmentation shows that the sensor-based detection is not influenced by the sensor location nor by the exercise performed.

### 3.2. Joint Stride Detection

The validation of the gait parameters computed from the inertial data is performed by comparing them to the ones measured by the reference system, optical motion capture, in our study. Consequently, only strides that have simultaneously been detected by both the inertial and optical motion capture systems can be used to compute the gait features on and, subsequently, compare them.

**Figure 9:**
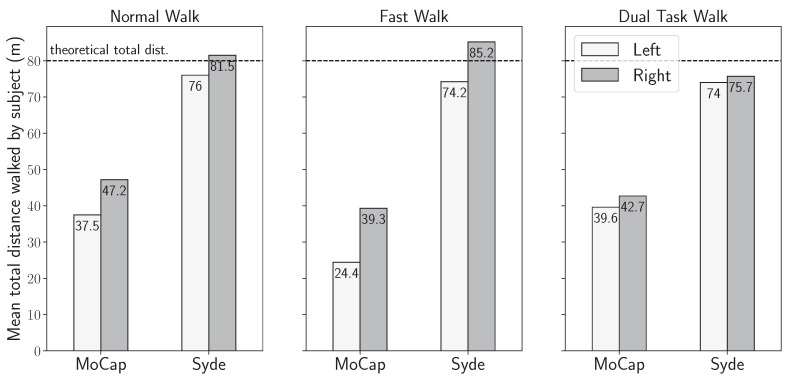
Mean total distance walked by subject, in meters, by side, and by exercise.

The first step was to compare the stride detection performance of both systems. To perform this, Figure 9 shows the average distance of the twelve participants for the three 80-m exercises (normal speed, fast walk, and dual-task), computed from the total stride length with both systems.

The average distance measured with the IMU-based wearable was between 74 m (left foot of the dual-task walking test) and 85 m (right foot of the fast walking test), as shown in Figure 9. A slight but systematically shorter distance was observed on the left side compared to the right foot. With the motion capture system, the average distance traveled was between 24.4 m (left foot of the fast walking test) and 42.7 m (right foot of the normal walk test). This was half the distance traveled by the participants. A shorter distance on the left side was also observed on the three walking test configurations. The stride detection with the IMU system is close to the 80 m distance defined in the protocol. However, the motion capture system only detects half the distance. This difference will be discussed in Section 4 of this paper. In the rest of the analysis, only the strides detected by the two systems are included in the gait features computation.

**Figure 10:**
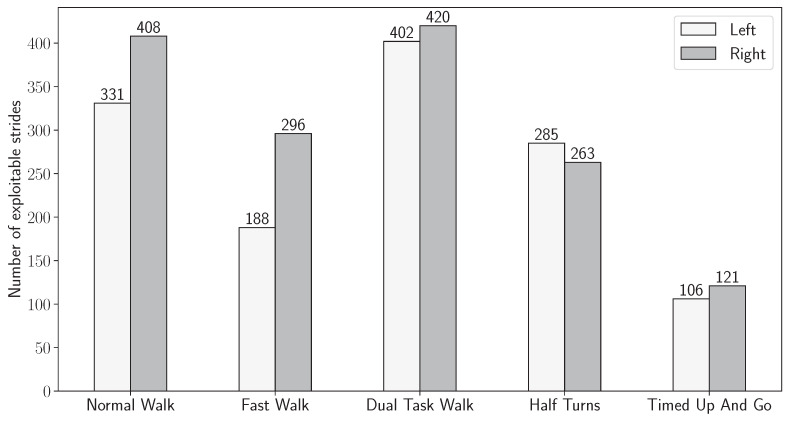
The number of strides recovered from inertial data and motion capture data for each exercise and side of the ankle where the devices are worn. Strides are used when data are available on both devices simultaneously.

Figure 10 finally shows the histogram of the number of strides detected for each foot for all participants per exercise.

A total of 2820 strides were detected by both systems, of which 1312 strides were taken by the left foot and 1508 by the right foot. As shown in Figure 9, an asymmetry remains between the left and right feet. Nevertheless, all the 2820 strides are included in the following stages of the analysis, and the observed asymmetry is discussed in Section 4. Since we have shown in the previous subsection that the inertial-based stride segmentation displayed a performance good enough to consider most strides to be successfully detected, the missed strides in the joint distribution are, therefore, imputed to defects in the motion capture acquisition, which limitations will also eventually be discussed in Section 4.

### 3.3. Gait Features Distribution in Both Systems

**Figure 11:**
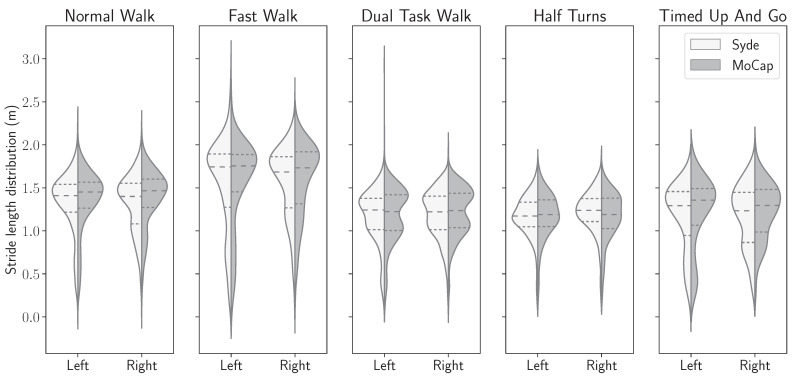
The stride length distributions, computed from the inertial sensor and the motion capture ground truth, for each ankle, for all five exercises.

**Figure 12:**
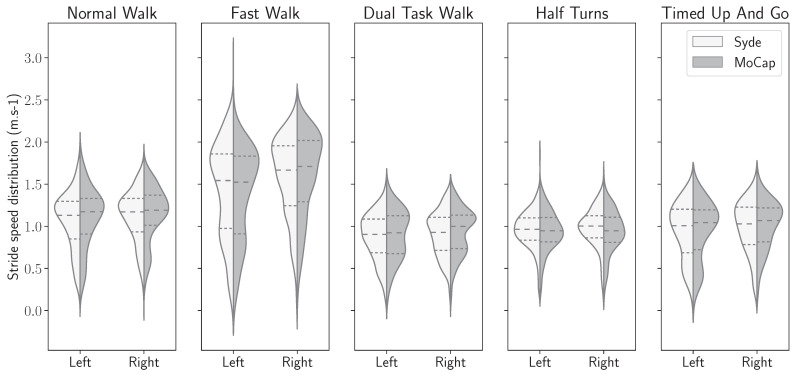
The horizontal stride velocity distributions, computed from the inertial sensor and the motion capture ground truth, for each ankle, for all five exercises.

In this subsection, the stride length and horizontal gait velocity features with both systems were computed. The densities of the statistics distributions for both features on each foot and each exercise are shown in Figure 11 and Figure 12.

In Figure 11, we observe most stride values on the normal walk exercise are between 1.0 and 2.0 m. As expected, the strides realized during the fast walk exercise are longer, with values ranging from 1.2 m to 2.5 m, and shorter for all the three remaining exercises; the values ranging in those cases between 0.7 m and 1.8 m for the dual-task and half-turns exercises and 0.5 m to 1.8 m for the timed-up-and-go exercise.

As for the horizontal stride velocity distributions shown in Figure 12, there is also evidence of matching distributions for all five exercises. The stride velocity distribution ranges from 0.5 to 1.9 m per second for the normal walk exercise. Concordantly, the values are higher for the fast walk exercise, with values that spread between 0.5 and 2.5 m per second. Similarly, the velocity distributions are lower for the three remaining exercises, with the values ranging between 0.3 and 1.5 m per second.

Lastly, for each foot and each exercise, as shown in Figure 11 and Figure 12, there is a similar distribution of median, quartiles, and spread density between the IMU-based wearable and the motion capture system. This indicates that the stride length and horizontal stride velocity characteristics calculated by the IMU-based wearable are similar to those calculated by the reference system.

### 3.4. Precision of Motion Capture Reference

The calibration performed in our study was satisfactory with respect to the manufacturer’s guidelines [33], which pledged a maximum mean residual error of 0.8 mm for the calibration. The precision obtained in our calibration was 0.789 mm and is, therefore, under the aforementioned threshold. This value, when compared with the mean stride length reconstructed by the motion capture reference of 1.3077 m, represented a mean error rate of 0.06%. The maximum residual error was 2.94 mm, representing a maximal error of 0.22% for the positioning precision.

### 3.5. Gait Features Error Distribution

Considering the left and right stride detection asymmetry shown in Figure 9, we state the hypothesis that both feature error distributions are the same on the left ankle and on the right ankle, and we analyze both acquisition methods on the global set of the 2820 strides, rather than separately.

To that extent, we performed a two-sided Mann–Whitney U-test. For both features, the tests on the error distribution for the normal walk, fast walk, and half-turns exercises yielded a *p*-value above the 0.05 threshold and, therefore, were not statistically significant (*p*-value > 0.05). In contrast, for both features, the test on the dual-task and the timed-up-and-go exercises produced a *p*-value below that fixed threshold and were statistically significant (*p*-value < 0.05). When computed on the global set of strides, for all exercises, the *p*-value was above the threshold (*p*-value > 0.05). We concluded not to reject the null hypothesis and to consider that the left and right strides distributions are similar, with the caveat that extra caution should be taken when concluding upon the cases of the exercises that were statistically significant for the hypothesis test.

**Figure 13:**
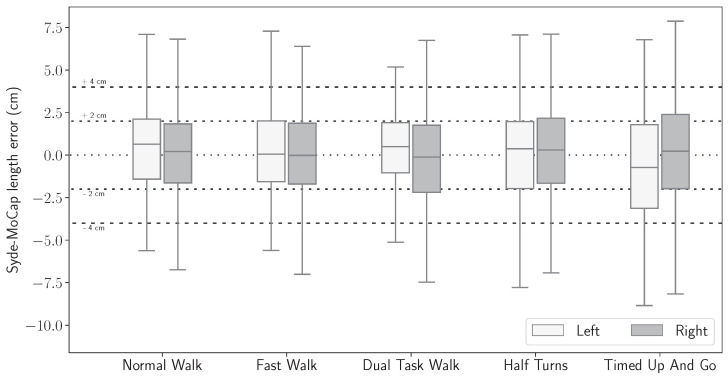
Stride length signed error distribution, computed between the IMU-based wearable reconstructed trajectory and the motion capture ground truth, by exercise.

**Figure 14:**
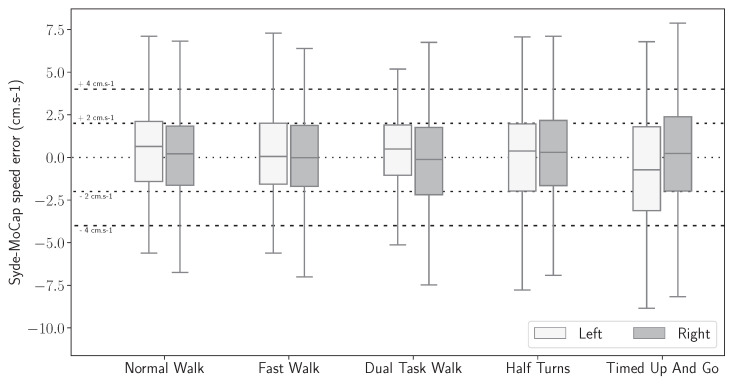
Stride velocity signed error distribution, computed between the IMU-based wearable reconstructed trajectory and the motion capture ground truth, by exercise.

Figure 13 and Figure 14 present the statistical distributions of the difference between stride length and horizontal velocity computed with the IMU-wearable system and the motion capture system.

Figure 13 and Figure 14 show that the mean difference between the inertial motion reconstruction and the optical motion reconstruction is centered around zero for both features and for all exercises. Moreover, in Table 2, an overall median of 1.86 cm and the 75th percentile at 3.24 cm were computed for the stride length error. The intra class correlation (ICC two-way-random: ICC2k) was statistically analyzed on all detected strides. The computed correlation was excellent (ICC2k > 0.99, CI95 [0.99, 0.99]).

Similarly, stride velocity error distribution was computed with a median at 1.56 cm/s and 75th percentile at 2.63 cm/s, as shown in Table 3. The correlation was also excellent (ICC2k, >0.99, CI95 [1.0, 1.0]).

**Figure 15:**
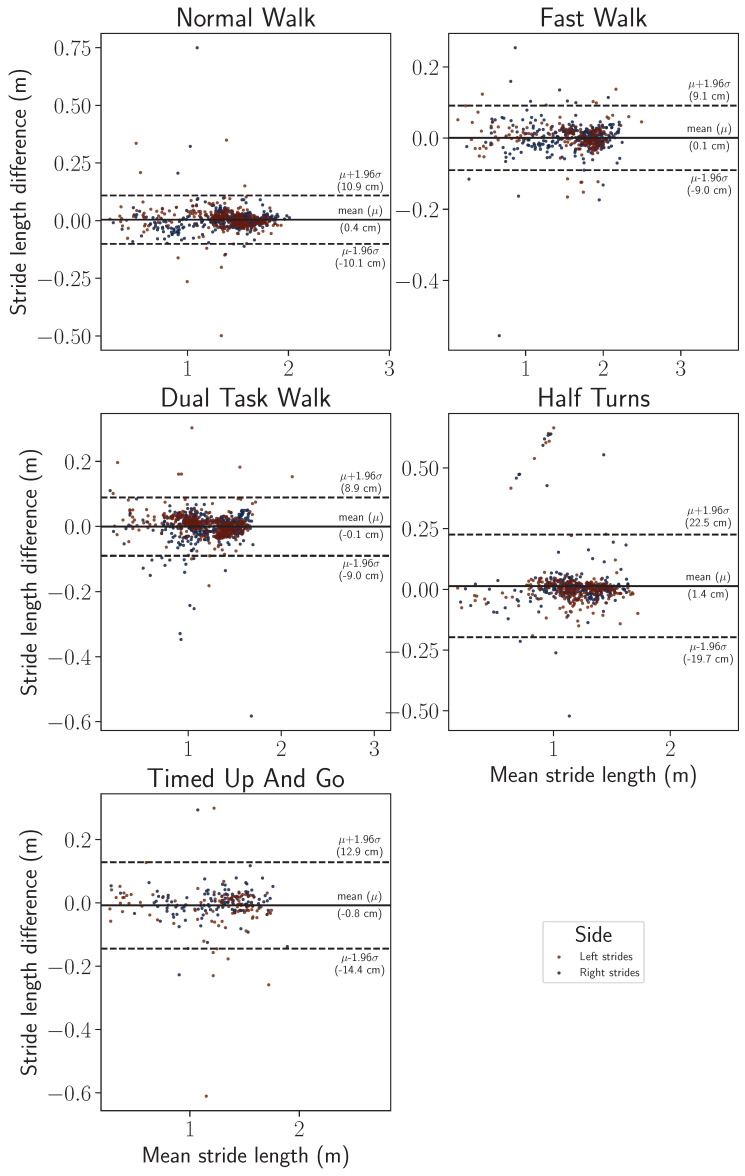
Bland–Altman comparison (IMU-based wearable versus MoCap reference) for the stride length feature by exercise, differentiated by wearing side.

**Figure 16:**
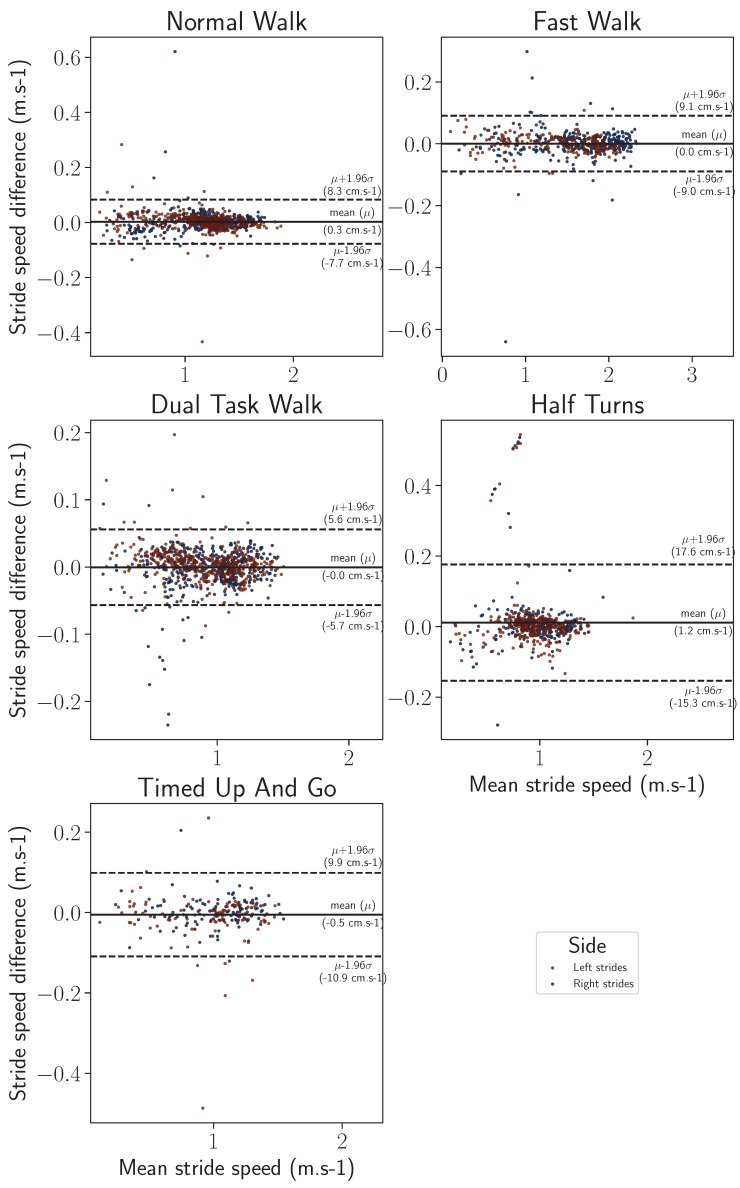
Bland–Altman comparison (IMU-based wearable versus MoCap reference) for the stride velocity feature by exercise, differentiated by wearing side.

**Figure 17:**
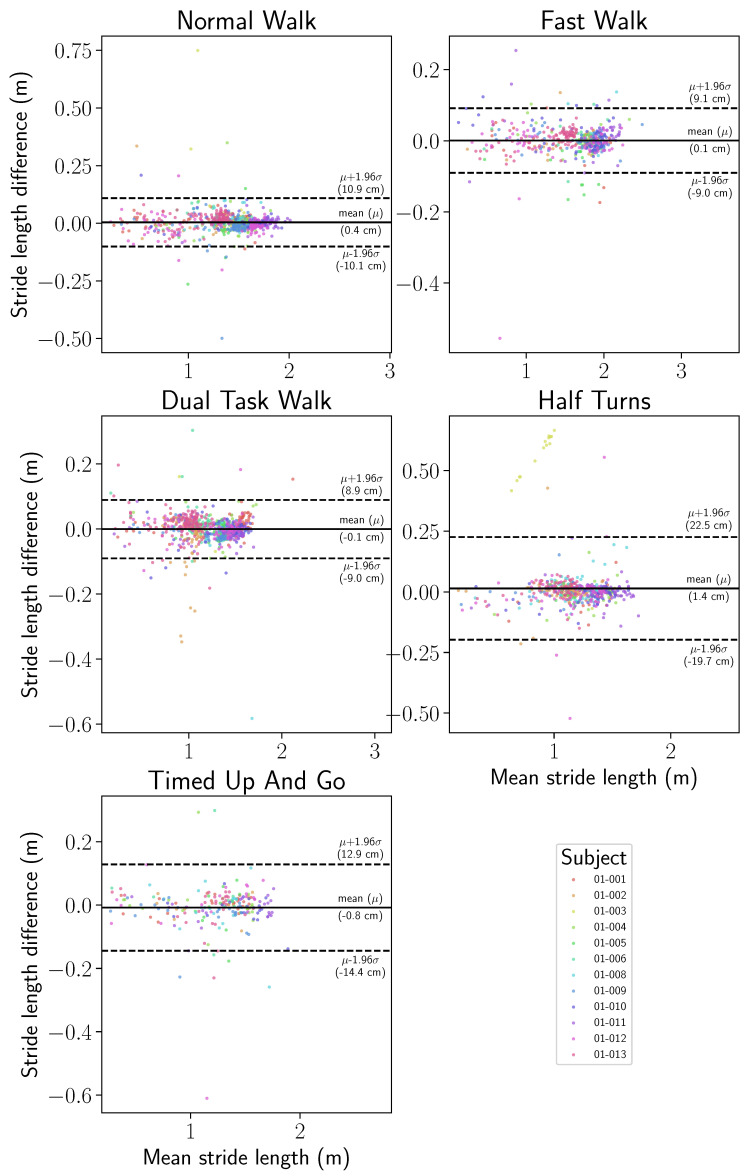
Bland–Altman comparison (IMU-based wearable versus MoCap reference) for the stride velocity feature by exercise, differentiated by subject.

**Figure 18:**
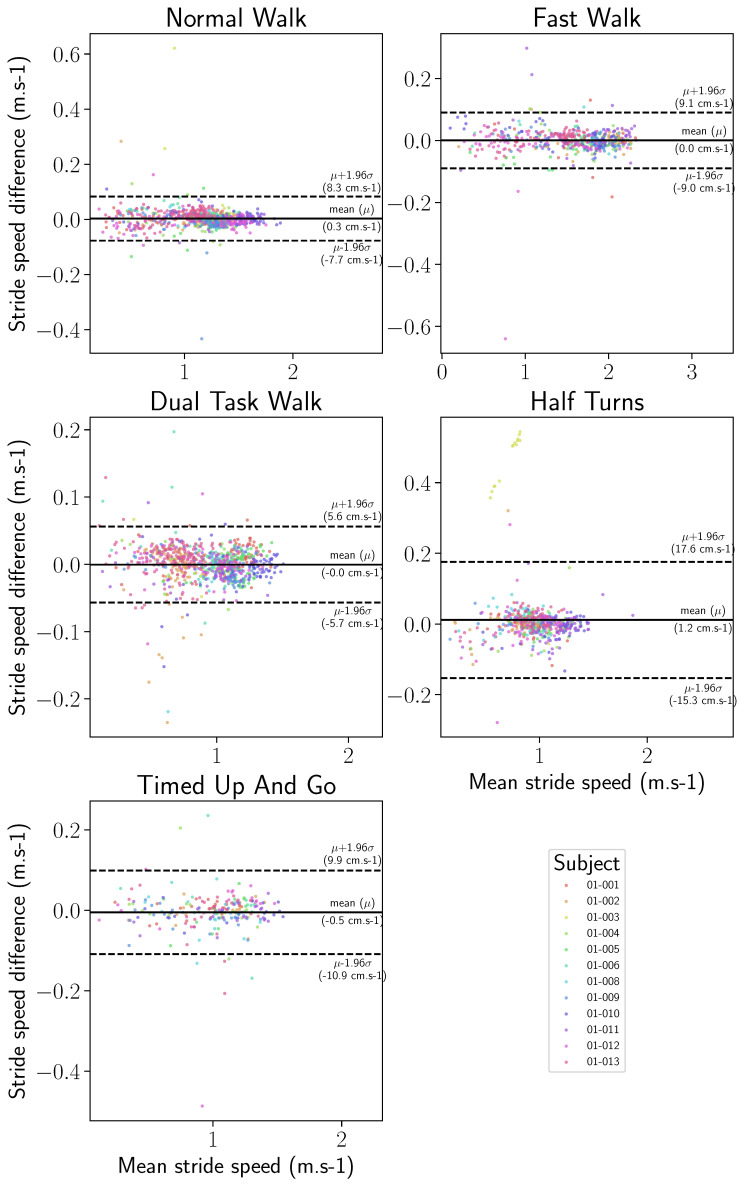
Bland–Altman comparison (IMU-based wearable versus MoCap reference) for the stride velocity feature by exercise, differentiated by subject.

Finally, Bland–Altman visualizations show, for each of the two gait parameters, the variance of the stride length and horizontal stride velocity estimations, as well as the variance of the inertial reconstructions, with respect to the motion capture ground truth. Such visualizations are provided with a split by wearing side in Figure 15 and Figure 16 and by participant in Figure 17 and Figure 18. On all the exercises except for the half-turns where a bias equal to 1.4 cm appears for the stride length and 1.2 cm·s^−1^ for the horizontal stride velocity. This indicates that the inertial reconstruction suffers from little to no systematic bias for four out of the five exercises, and an average bias representing around 1% of the stride length or speed for the half-turns exercise. These results are not influenced by the wearing side or the participants who produced the analyzed strides, except for the specific case of the subject 01–003 in the half-turns exercise, which can be considered an outlier. In addition, for both features, the bias does not change with the value of the feature. Lastly, very few strides stand outside the error band μ±σ, with μ as the empirical mean error of the feature of interest, and σ as the sample standard deviation. This evidences the low variance of the inertial reconstruction method compared with the motion capture reference.

## 4. Discussion

In this study, the IMU-based wearable Syde^®^ analytical algorithm gait parameters, stride length and mean horizontal velocity over a stride, were compared to the same features computed from the gold standard motion capture system on five different walk exercises (normal walk, fast walk, double task walk, turns, timed-up, and go) on 12 healthy subjects.

**Figure 19:**
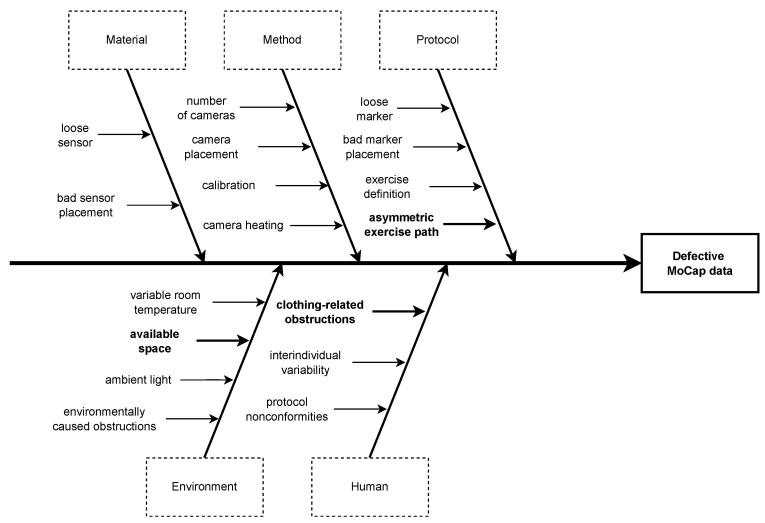
Root cause analysis of the defective motion capture data.

The first thing that needs to be discussed is the asymmetry in the stride detection between the two systems. The mean total reconstructed distance recovered shown in Figure 9 is significantly less for the strides recovered and analyzed from the motion capture data than for the IMU-wearable-obtained strides. The inertial-based stride detection validation that has been performed beforehand, and which results are summarized in Figure 8, shows that the motion capture is the limiting factor for the gait features analysis, as it only allows for retrieving half the maximal expected distance, whereas 95.9% of the strides are successfully segmented by the sensor-based detection. The theoretical total distance walked by each subject is expected to be around 80 m in the case of a six-loop walk around the track defined by the protocol. However, the subjects tended to move away from the inside of the track proportionally to their walking pace, with the latter being lowest for the dual-task walk and the highest for the fast walk. Therefore, the actual total distances vary with the same logic. As for the motion capture strides, the motion capture acquisition is less efficient when the subjects walk at a higher pace (Figure 19), with a lesser number of detected strides compensating the estranging effect previously evidenced.

Another important point to discuss is the asymmetry between left and right feet in the 2820 strides detected by both systems and included in the analysis. The remaining asymmetry between the right and left foot observed could be explained by the asymmetric exercise path where the left foot is always oriented toward the interior of the walking track. The right foot is outside the curve; the simple physical reason for this asymmetry is that the right foot covers a higher distance than the left. Figure 8 again shows that the inertial-based stride detection does not manifestly suffer from asymmetry matters. Also, as the motion capture cameras are placed outside the exercise track, the occasional occlusion of the left-ankle reflective markers by the right ankle can happen during an acquisition run. Making this test per exercise does not make much sense as the typology of gait patterns varies too much between the strides performed with the left foot and the strides performed with the right foot. Indeed, with the shape of the walking path being oval, left strides are logically closer to the inner part of the trajectory, especially in the turns. Consequently, the feature’s stride length and stride mean horizontal velocity aggregation were performed on each foot independently and not on an average of the feet. The asymmetry thus has no impact on the results shown here. This difference can be explained by the speed at which the three exercises taken into consideration were realized: the dual-task walk was performed at a slower pace than the normal walk exercise, itself, in essence, realized slower than the fast walk exercise. When the round-trip is performed at a higher speed, the subjects tend to take the bend wider and stray away from the interior of the curve, finally making them walk a higher overall distance.

Despite this slight asymmetry, similar distributions of stride length were observed on the wearable device computed and motion capture data (Figure 11), with an overall median difference below 2 cm (1.86 cm), and a 75th percentile of 3.24 cm (Table 2). The intra class correlation was excellent between IMU-wearable-based gait features and the reference system (ICC(2,1) > 0.99, CI95% [0.99, 0.99]). Similar results were observed with stride mean horizontal velocity. There was a similar statistical distribution across the IMU-based wearable analytical algorithm and the motion capture system computation (Figure 12). The computed median was 1.56 cm·s^−1^ and a 75th percentile of 2.63 cm·s^−1^ (Table 3). ICC was also computed, and there was an excellent correlation between the two methods (ICC2k, >0.99, CI95 [1.0, 1.0]). We also observed a repartition centered on a zero value that we interpret as an absence of systematic bias from one measurement method compared to the other one (Figure 13, Figure 14, Figure 15 and Figure 16). These results support the initial hypothesis that the computed features of stride length and stride mean horizontal velocity with the IMU-based wearable device analytical algorithm are similar to those computed on the motion capture system.

This study presents some limitations that we group into three main categories.

One of the challenges has been finding a balance between the plurality of exercises and environmental restrictions caused by the limited available space (Figure 3, Figure 4 and Figure 5). The diversity of exercises aims to gather a broad range of gait patterns (initiation stride, half-turns, fast walk, transition from sitting to standing up). Because of the small area, we had a slight imbalance of half-turns, acceleration, and deceleration strides compared to regular strides. As mentioned before, the asymmetry of walk exercises may be an issue; indeed, in the first three exercises, participants always turn in the same direction, with the left foot on the interior side. This has probably contributed to the occlusion of the MoCap markers observed on the left foot and then stride detection with the MoCap algorithm. Creating a symmetrical eight-shaped walking path would be one solution to alleviate the occurrence of occlusions and hinder participants from drifting out of the camera field, narrowing the turning angle.

Because of environmental conditions, human-related factors, and protocol design flaws with the asymmetric path (Figure 19), we had some noisy motion capture data that we could not include in the analysis and ended up with less stride detected on the left foot.

Finally, the participant bias of selection is one limitation of our work. Indeed, our study includes healthy and mostly young male subjects (mean age of 23 years old). Given the natural evolution of gait in aging, validation with healthy middle-aged and elderly individuals is needed. Moreover, gait patterns are altered depending on the localization of the affection. Validation should also be performed with patients suffering from different gait alteration affections.

## 5. Conclusions

This article presents the analytical validation of the stride length and horizontal velocity features extracted from the Syde^®^ wearable sensor in comparison to a reference motion capture system, with the participation of 12 healthy participants. For this analytical validation, a total of 2820 strides were computed from five different walking exercises performed by each participant. Among these strides, the median absolute error between the reference system and the reconstructed stride was 1.86 cm for stride length and 1.56 cm/s for stride horizontal velocity.

With a median measurement difference with the gold standard of less than 2 cm, a 75th percentile of less than 4 cm for the total stride length characteristics, a median horizontal stride velocity of less than 2 cm/s, and a 75th percentile of less than 3 cm/s, the present results demonstrate the accuracy of these two temporal and spatiotemporal gait parameters, respectively, computed with the Syde ^®^ IMU-based wearable device. We conclude with an analytical validation of the computation algorithm of these two features.

The features extracted from IMU-based wearable devices must be robust and rigorously validated to be used in clinical research. The results presented in this study support that the IMU-based wearable Syde^®^ device computed features of stride length and horizontal stride velocity are accurate enough to be used in a real-world setting in a free-living environment on healthy subjects. Further studies with patients with different types of conditions in a controlled environment are needed to investigate the accuracy of these features in altered gait. 

## Figures and Tables

**Table 1:** Details of participant demographic data, exercises performed, and available data for IMU-based wearable device motion capture comparison.

Subject	Gender	Age (years)	Height (cm)	Weight (kg)	BMI (kg·m^−2^)
01	M	23	181.0	63	19.2
02	M	25	178.0	72	22.7
03	M	24	175.0	72	23.5
04	M	22	174.5	70	23.0
05	M	27	179.0	86	26.8
06	F	22	165.0	52	19.1
07	M	22	180.0	65	20.1
08	M	24	174.0	60	19.8
09	F	21	164.5	55	20.3
10	M	26	176.0	68	22.0
11	M	21	180.0	63	19.4
12	F	21	163.0	55	20.7

**Table 2:** Absolute stride length error distributions between the inertial sensor acquisition and the motion capture ground truth (NW: normal walk, FW: fast walk, DTW: dual-task walk, HT: half-turns, TUG: timed up-and-go).

Sensor Location	Left Ankle			Right Ankle			Overall		
**Exercise**	**Q1**	**Median**	**Q3**	**Q1**	**Median**	**Q3**	**Q1**	**Median**	**Q3**
NW (cm)	0.93	1.87	3.23	0.86	1.73	2.99	0.88	1.81	3.11
FW (cm)	0.93	1.72	3.01	0.86	1.81	3.05	0.87	1.79	3.03
DTW (cm)	0.69	1.55	2.95	0.98	1.98	3.20	0.83	1.74	3.05
HT (cm)	1.10	1.97	3.67	0.88	1.92	3.98	0.94	1.95	3.82
TUG (cm)	1.07	2.33	4.04	1.08	2.17	3.70	1.07	2.29	3.77
**Overall (cm)**	0.87	1.84	3.14	0.89	1.90	3.32	0.88	**1.86**	3.24

**Table 3:** The absolute horizontal stride velocity error distributions between the inertial sensor acquisition and the motion capture ground truth. (NW: normal walk, FW: fast walk, DTW: dual-task walk, HT: half-turns, TUG: timed up-and-go).

Sensor Location	Left Ankle			Right Ankle			Overall		
**Exercise**	**Q1**	**Median**	**Q3**	**Q1**	**Median**	**Q3**	**Q1**	**Median**	**Q3**
NW (cm·s^−1^)	0.77	1.50	2.45	0.74	1.50	2.57	0.75	1.50	2.51
FW (cm·s^−1^)	0.77	1.58	2.69	0.88	1.78	3.05	0.84	1.71	2.87
DTW (cm·s^−1^)	0.54	1.21	2.09	0.77	1.55	2.48	0.64	1.38	2.26
HT (cm·s^−1^)	0.83	1.65	2.81	0.70	1.59	3.32	0.77	1.61	3.06
TUG (cm·s^−1^)	0.92	1.84	2.74	0.89	1.77	3.08	0.90	1.84	3.01
**Overall (cm·s^−1^)**	0.71	1.48	2.47	0.77	1.61	2.75	0.74	**1.56**	2.63

## Data Availability

The data that support the findings of this study are available upon request from the corresponding authors, A.G. and M.M. The data are not publicly available due to their containing information that could compromise the privacy of the research participants.
